# SARS‐CoV‐2 ORF6 protein targets IFP35 to inhibit the IFNγ signaling pathway

**DOI:** 10.1002/mlf2.70112

**Published:** 2026-10-26

**Authors:** Lihong Liu, Jie Du, Jiacheng Huang, Sidi Yang, Qiaoqiao Xiao, Liu Cao, Hong Peng, Birong Zheng, Chunmei Li, Deyin Guo

**Affiliations:** ^1^ Guangzhou National Laboratory Guangzhou China; ^2^ Centre for Infection and Immunity Studies (CIIS), School of Medicine Shenzhen Campus of Sun Yat‐sen University Shenzhen China; ^3^ Guizhou University of Traditional Chinese Medicine Guiyang China

**Keywords:** IFNγ, IFP35, ORF6, SARS‐CoV‐2, type II interferon

## Abstract

The innate immune system functions as the frontline barrier against invading pathogens, such as severe acute respiratory syndrome coronavirus‐2 (SARS‐CoV‐2). Nevertheless, the virus has developed various strategies to effectively regulate host immune responses, particularly through the inhibition of interferon signaling. In this study, we identify the SARS‐CoV‐2 accessory protein ORF6 as a key inhibitor of interferon gamma (IFNγ) signaling through downregulation of the interferon‐induced 35‐kDa protein (IFP35). *IFP35*, an interferon‐stimulated gene (ISG) critical for interferon ‐mediated antiviral response, is significantly upregulated during SARS‐CoV‐2 infection. Silencing IFP35 via RNA interference enhances viral replication, indicating its antiviral role. Furthermore, we demonstrate that ORF6 associates with IFP35 and accelerates its ubiquitin–proteasome degradation, thereby impairing IFP35's ability to activate IFNγ signaling. These data reveal novel mechanisms used by SARS‐CoV‐2 to circumvent host immunity, specifically by impairing IFNγ signaling.

## Impact statement

The mechanism underlying SARS‐CoV‐2's suppression of type II interferon (IFNγ) responses has remained elusive. Here, we uncover that ORF6—a viral accessory factor—exerts its immunosuppression function through targeted downregulation of IFP35. Mechanistically, ORF6 binds IFP35 and facilitates its ubiquitination‐dependent turnover, thereby suppressing IFP35‐mediated activation of IFNγ signaling and subsequent antiviral defense mechanisms. Our findings provide novel mechanistic insights into SARS‐CoV‐2's immune escape and unveil a promising therapeutic target against COVID‐19.

## INTRODUCTION

Severe acute respiratory syndrome coronavirus‐2 (SARS‐CoV‐2), an enveloped, positive‐sense, single‐stranded RNA virus within the family *Coronaviridae*, is responsible for coronavirus disease 2019 (COVID‐19)^1^, ^2^, ^3^. This pathogen has led to millions of deaths and substantial morbidity worldwide^4^. The advent of effective vaccines and antiviral agents has led to a marked decline in severe COVID‐19 outcomes. Nevertheless, the disease persists as a widespread and life‐threatening illness, thereby impeding ongoing attempts to mitigate its global burden. This is primarily attributed to the unique pathogenesis of the virus and the complexities of host immunological defense.

The innate immune system functions as the primary barrier against pathogens, such as SARS‐CoV‐2^5^, ^6^, ^7^. Nevertheless, SARS‐CoV‐2 has acquired adaptive mechanisms to bypass immune recognition and amplify its replication and spread. Immune responses elicited by SARS‐CoV‐2 differ substantially from those induced by other respiratory viruses^7^, ^8^. Studies have shown a complete absence of or substantial delay in type I (IFN‐I) and type III (IFN‐III) interferon signaling in patients, cell lines, and animal models infected with SARS‐CoV‐2, pointing to dysregulated innate immunity^9^. In contrast, the contribution of type II interferon (IFN‐II), particularly IFN‐gamma (IFNγ), remains ambiguous. As the sole member of the IFN‐II family, IFNγ plays a key role in host defense against pathogens by promoting antigen presentation via MHC class I and class II molecules, directing cell fate decisions, activating lymphocytes, and modulating apoptosis, as well as stimulating or repressing the expression of numerous genes^10^, ^11^. A recent study demonstrated that all types of IFNs, including IFN‐II, show antiviral efficacy when administered shortly after viral exposure^12^. Another study showed that IFNγ treatment slightly reduced viral replication, whereas its co‐administration with IFN‐β resulted in a pronounced reduction in SARS‐CoV plaque formation and viral load^13^. Therefore, advancing our understanding of how type II IFN responses function during SARS‐CoV‐2 infection is pivotal for guiding the design of successful therapeutic interventions.

While SARS‐CoV‐2 accessory proteins are not required for virus replication, some of these proteins exert a critical influence on host–pathogen crosstalk and pathogenesis^14^. ORF6, an accessory protein highly conserved among sarbecoviruses, including SARS‐CoV and SARS‐CoV‐2, functions as a potent type I IFN antagonist. It efficiently blocks STAT1 nuclear translocation by directly binding to Rae1‐Nup98 via its C‐terminal domain^15^, ^16^, ^17^. Furthermore, ORF6 sequesters Rae1 on cytoplasmic membranes, disrupting the shuttling of mRNA between the nucleus and the cytoplasm^18^. ORF6 has also been linked to the DNA damage response elicited by SARS‐CoV‐2, acting via CHK1 degradation and disruption of 53BP1 recruitment at DNA lesion sites^19^. Most recently, ORF6 has been shown to disrupt the STAT1–IRF1–NLRC5 signaling cascade, thereby dampening type II interferon‐mediated activation of the MHC class I pathway^20^.

Here, we report that the viral protein ORF6 from SARS‐CoV‐2 suppresses IFNγ signaling by downregulating IFP35—a 35‐kDa interferon‐stimulated protein. IFP35, an interferon‐stimulated gene (ISG) family member, is induced by IFNγ and plays a role in antiviral defense^21^. We observe a marked increase in IFP35 levels upon SARS‐CoV‐2 exposure, and knockdown of IFP35 enhances viral replication. Mechanistically, ORF6 binds IFP35 directly, triggering its ubiquitin–proteasome degradation, thereby suppressing IFP35‐induced IFNγ signaling. Our findings enhance the understanding of immunopathogenic mechanisms of SARS‐CoV‐2 and inform potential targeted interventions for treating COVID‐19.

## RESULTS

### IFNγ signaling response is augmented during SARS‐CoV‐2 infection

To assess the impact of SARS‐CoV‐2 on the type II interferon pathway, we re‐analyzed an RNA‐seq cohort derived from lung cells infected with the virus (GSE147507)^8^. In both Calu‐3 and A549‐ACE2 cells, the IFNγ response axis was robustly engaged in infected cells, with a marked transcriptional upregulation of IFNγ‐associated genes (Figure 1A). Among the upregulated genes, the mean expression of *IFP35* increased approximately fivefold under viral challenge (Figure 1B). In a separate dataset from autopsy specimens of COVID‐19 patients (GSE150316), differential expression of IFNγ response genes was observed to correlate with the viral RNA concentration. High viral RNA cases were enriched for elevated expression of IFNγ response genes, including *IFP35* ^22^. As shown in Figure 1C, *IFP35* expression in high viral RNA cases (*n* = 19) was significantly higher than that in low viral RNA cases (*n* = 33) and negative control cases (*n* = 5). These results indicated that IFNγ signaling was augmented, and the IFNγ‐stimulated gene *IFP35* was upregulated during SARS‐CoV‐2 infection.

**Figure 1:**
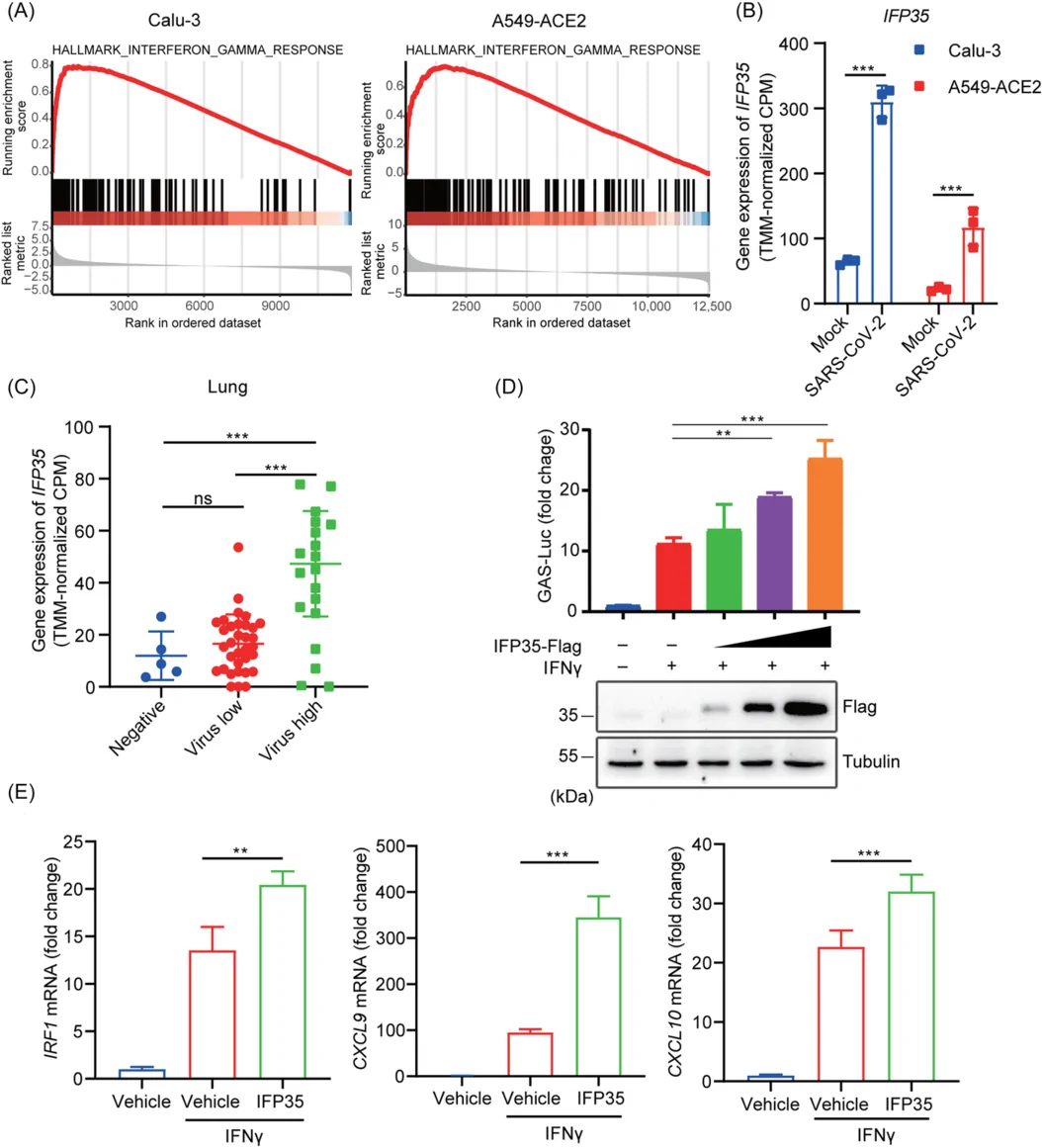
The interferon gamma (IFNγ) response is activated by SARS‐CoV‐2 infection and is augmented by IFP35. (A) Gene set enrichment analysis (GSEA) of the HALLMARK_INTERFERON_GAMMA_RESPONSE pathway in Calu‐3 and A549‐ACE2 cells. The *x*‑axis shows genes ranked by log_2_(fold change) (left: upregulated; right: downregulated). The top panel displays the running enrichment score (RES; red line), whose peak represents the enrichment score (ES). The middle panel indicates the position of pathway genes within the overall ranked list (black vertical bars). The red‑to‑blue gradient bar below reflects gene expression correlation with the phenotype (red: higher expression; blue: lower expression). The bottom panel (gray line) shows the distribution of the ranked list metric across all genes in the dataset. (B) Expression of *IFP35* induced by SARS‐CoV‐2 infection. *IFP35* expression levels were quantified as counts per million (CPM) using trimmed mean of M‐values (TMM)‐normalized data. Statistical analysis was performed with *edge*R using a Benjamini–Hochberg‐adjusted two‐sided Wald test. Data in (A, B) are re‐analysis results of data from Blanco‐Melo et al. (GSE147507)^8^, and data from cell lines Calu‐3 and A549‐ACE2 infected with SARS‐CoV‐2 were normalized against mock‐treated cells. ***p < 0.001. (C) Re‐analysis of the autopsy specimen dataset from patients who succumbed to SARS‐CoV‐2 infection (GSE150316)^21^. According to quantitative RNA‐ISH analysis, patients with a ratio of virus‐infected lung area to total lung area ≥2% were defined as “Virus high” and those with a ratio <2% were classified as “Virus low.” *IFP35* expression levels were quantified as CPM from RNA‐seq data derived from lung tissue samples, including “Virus high” (*n* = 19 samples from 6 cases), “Virus low” (*n* = 33 samples from 11 cases), and non‐COVID‐19 controls (*n* = 5). (D) Dose‐dependent enhancement of IFNγ‐induced gamma‐activated site (GAS) promoter activity by IFP35. HeLa cells were transfected with increasing doses of IFP35‐Flag for 24 h and then treated with IFNγ for 10 h. GAS‐Luc activity was determined by the dual luciferase reporter assay and the expression of IFP35‐Flag was verified by immunoblotting. (E) IFP35 amplifies the expression of IFNγ‐stimulated genes. HeLa cells were transfected with IFP35‐Flag for 24 h, followed by treatment with IFNγ for 10 h. The expression levels of the indicated genes were then analyzed by reverse transcription quantitative real‐time PCR (RT‐qPCR). (D, E) Data are from three independent experiments; comparisons in (E) were made with an unpaired, two‐tailed Student's *t* test and those in (D) with one‐way ANOVA. ns, not significant; **p < 0.01; ***p < 0.001.

In order to investigate the role of IFP35 in IFNγ signaling, we constructed a gamma‐activated site luciferase (GAS‐Luc) reporter plasmid to monitor the promoter activity of IFNγ‐mediated IRF1 GAS. The reporter assay system was validated in HeLa cells, where increasing doses of IFNγ treatment enhanced GAS‐Luc activity and increasing doses of ORF6 expression significantly inhibited IFNγ‐induced GAS‐Luc activity (Figure S1A–C). As expected, expression of IFP35 significantly promoted IFNγ‐mediated GAS‐Luc activity in a dose‐dependent manner (Figure 1D). To assess if IFP35 influences the expression of IFNγ‐induced genes, HeLa cells were transfected with IFP35 and subsequently stimulated with IFNγ. The expression of *IRF1*, *CXCL9*, and *CXCL10* was then analyzed by reverse transcription quantitative real‐time PCR (RT‐qPCR). IFP35 expression enhanced the IFNγ‐induced activation of these genes (Figure 1E), indicating that IFNγ signaling was augmented by IFP35. In contrast, IFP35 overexpression did not significantly augment IFN‐β‐induced interferon‐stimulated response element (ISRE)‐Luc reporter activity (Figure S1D), suggesting that its positive regulatory effect is selective for IFNγ signaling.

To determine whether IFP35 influences the core events of IFNγ signaling, we analyzed the effects of IFP35 silence on STAT1 activation and nuclear translocation. The results showed that *IFP35* knockdown did not significantly affect IFNγ‐induced STAT1 tyrosine phosphorylation (pY701) or its nuclear translocation (Figure S1E,F). These results suggest that IFP35 does not regulate the initial steps of IFNγ signaling; instead, it likely functions at the stage of STAT1‐mediated transcriptional activation, consistent with the role of N‐myc‐interactor (NMI)—a protein that shares structural similarity with IFP35 and belongs to the same family^21^, ^23^.

### IFP35 is upregulated during SARS‐CoV‐2 infection and inhibits viral replication

To validate the induction of IFP35 in response to SARS‐CoV‐2, two lung cancer cell lines—NCI‐H1299 and A549‐ACE2—were exposed to the virus. Subsequent RT‐qPCR corroborated RNA‐seq data from infected human tissues and cells, demonstrating a significant increase in *IFP35* mRNA expression in both infected cell lines (Figure 2A,B). These observations reinforce the conclusion that SARS‐CoV‐2 infection induces the upregulation of IFP35. In addition, we assessed *Ifp35* mRNA expression in lung tissue from SARS‐CoV‐2‐infected K18‐hACE2 transgenic mice. Notably, *Ifp35* mRNA in the lung tissue of infected mice was significantly elevated (Figure 2C).

**Figure 2:**
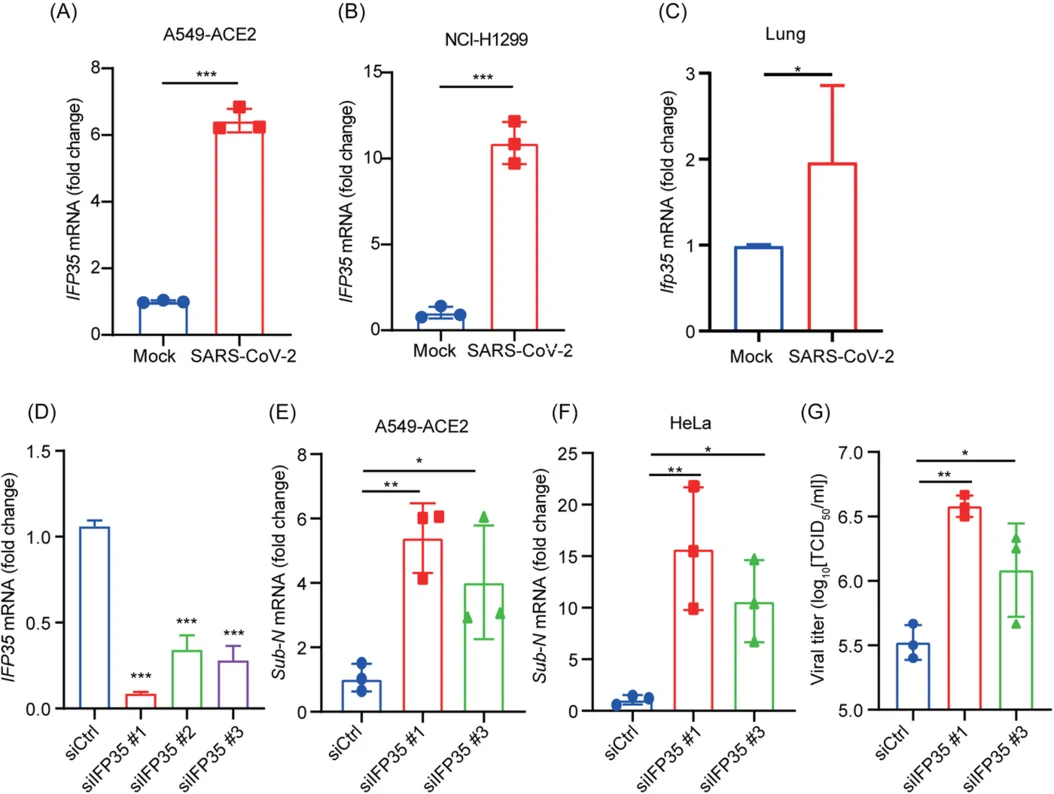
IFP35 expression is upregulated during SARS‐CoV‐2 infection and knockdown of *IFP35* promotes viral replication. (A, B) *IFP35* expression level elevated in SARS‐CoV‐2‐infected cells. A549‐ACE2 cells (A) and NCI‐H1299 cells (B) were infected with the SARS‐CoV‐2 Delta variant at an MOI of 0.05 for 48 h. *IFP35* mRNA levels were then analyzed by RT‐qPCR. (C) The mRNA level of *Ifp35* increased in the lung tissue of infected mice. K18‐hACE2 mice were intranasally inoculated with vehicle or with the SARS‐CoV‐2 Delta variant (*n* = 3). Lung tissues were harvested at 3 days post infection (dpi) and the expression level of *Ifp35* was analyzed by RT‐qPCR. (D) Validation of *IFP35* knockdown by siRNA. HeLa cells were transfected with siRNAs targeting *IFP35* for 48 h and then *IFP35* knockdown efficiency was confirmed by RT‐qPCR. (E, F) IFP35 knockdown enhances SARS‐CoV‐2 subgenomic N (*Sub‐N*) mRNA levels. A549‐ACE2 cells (E) and HeLa cells (F) were transfected with siIFP35 #1 and #3 for 48 h, and then infected with the SARS‐CoV‐2 Delta variant. Subgenomic RNA of SARS‐CoV‐2 was subsequently analyzed by RT‐qPCR. (G) *IFP35* knockdown increases infectious viral titers. A549‐ACE2 cells were transfected with siIFP35 #1 and #3 for 48 h, followed by infection with the SARS‐CoV‐2 Delta variant at an MOI of 0.05. At 48 h post infection, culture supernatants were collected and viral titers were determined by the TCID_50_ assay in Vero‐E6 cells. Data represent mean ± SD of three independent experiments. Comparisons between two groups (A–C) were made with an unpaired, two‐tailed Student's *t* test, and comparisons among three or four groups (D–G) with one‐way ANOVA. *p < 0.05; **p < 0.01; ***p < 0.001.

To evaluate its function in SARS‐CoV‐2 pathogenesis, we synthesized small interfering RNAs (siRNAs) targeting *IFP35*. Three independent siRNAs achieved efficient depletion of *IFP35* mRNA (Figure 2D). We then knocked down *IFP35* with siRNAs and infected the cells with SARS‐CoV‐2. It was shown that effective knockdown of *IFP35* led to enhanced viral replication, as demonstrated by increased viral RNA levels (Figure 2E,F) and a higher titer of infectious virus particles measured by the TCID_50_ assay (Figure 2G). These results suggest that IFP35 inhibits SARS‐CoV‐2 replication.

### SARS‐CoV‐2 ORF6 interacts with IFP35

Given that *IFP35* mRNA levels are upregulated following SARS‐CoV‐2 infection as part of the host antiviral response, we investigated whether SARS‐CoV‐2‐encoded proteins contribute to IFP35‐induced IFNγ signaling. A previous study showed that the NMI/IFP35 domains (NIDs) of NMI bind to SARS‐CoV ORF6^24^. Based on the sequence homology between SARS‐CoV and SARS‐CoV‐2, as well as between IFP35 and NMI, we hypothesized that SARS‐CoV‐2 ORF6 may interact with IFP35. To test this, we ectopically expressed ORF6 and IFP35 in HEK293T cells and conducted co‐immunoprecipitation (Co‐IP) assays. As shown in Figure 3A,B, IFP35 and ORF6 co‐precipitated with each other. Additionally, immunofluorescence microscopy revealed that IFP35 strongly colocalized with ORF6 (Figure 3C). The IFP35 protein comprises an N‐terminal leucine zipper (l‐Zip, 5–32 aa) and two tandem NID domains (NID1, 80–170 aa; NID2, 177–268 aa)^25^. To identify the specific regions responsible for the IFP35–ORF6 interaction, we constructed a series of IFP35 truncations, as shown in Figure 3D. Co‐IP assays indicated that simultaneous deletion of both NID1 and NID2 domains abolished the interaction with ORF6, while deletion of individual domains partially impaired the association between IFP35 and ORF6 (Figure 3E). These results suggest that both NID1 and NID2 domains contribute to the interaction between IFP35 and ORF6, and that these domains cooperate for maximal binding. In summary, these data reveal that ORF6 associates with IFP35, and that the NID domains within IFP35 are crucial for this association. To further identify the regions of ORF6 required for its interaction with IFP35, we performed Co‐IP assays using ORF6 mutants: M1 (49–52 aa substituted with alanines), M2 (53–55 aa substituted with alanines), and M3 (56–61 aa substituted with alanines) (Figure S2). As shown in Figure 3F, ORF6 M2 and M3 mutants showed significantly reduced binding to IFP35 compared to wild‐type ORF6 and the M1 mutant. Our data suggest that the C‐terminal region of ORF6, specifically the residues spanning amino acids 53–61, is critical for its interaction with IFP35, consistent with its known role in immune antagonism.

**Figure 3:**
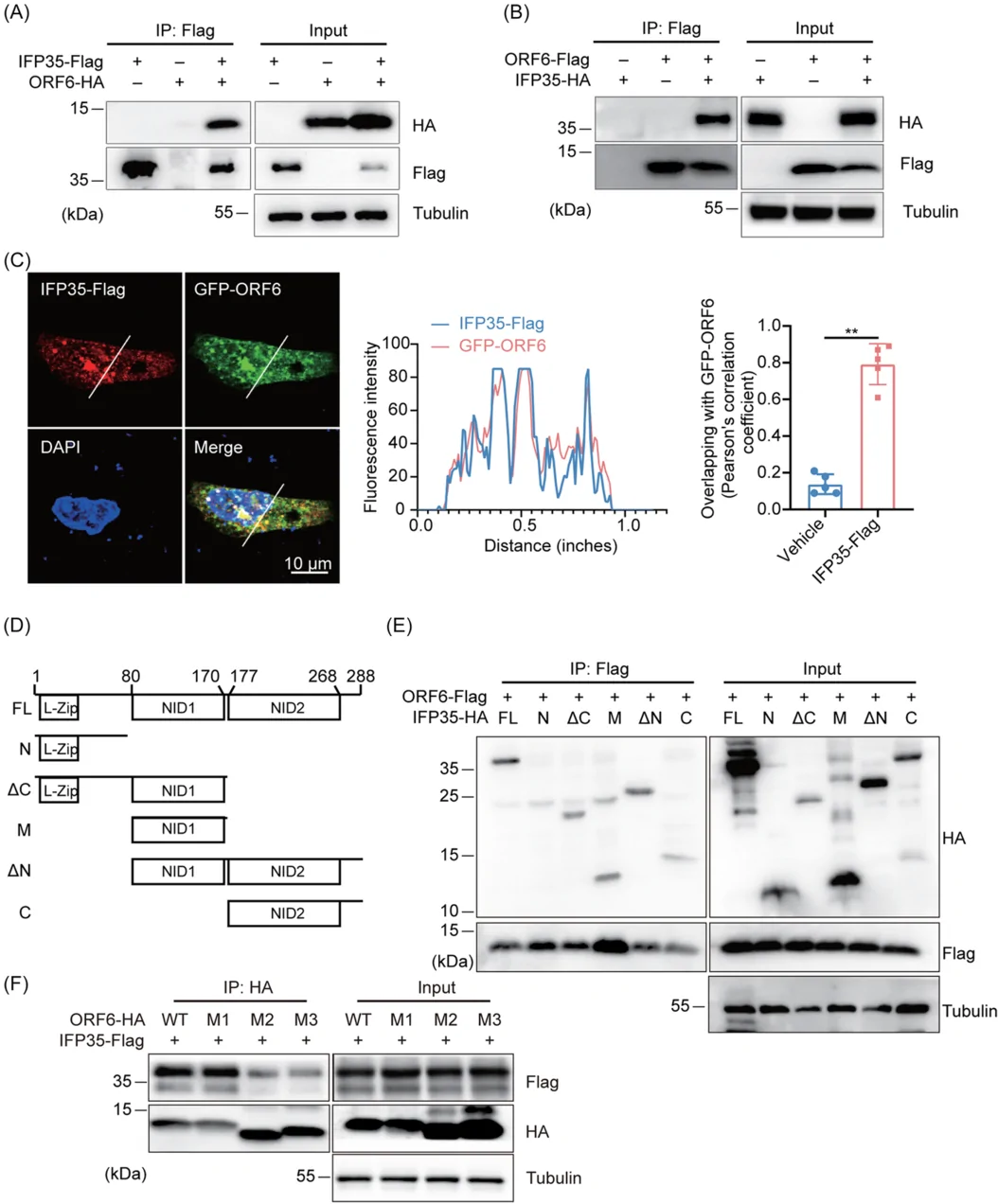
ORF6 interacts with IFP35. (A, B) Co‐immunoprecipitation (Co‐IP) between IFP35 (A) and ORF6 (B). HEK293T cells were transfected with the indicated plasmids for 24 h, followed by treatment with bortezomib (BTZ) for 10 h. Whole‐cell lysates were immunoprecipitated with anti‐Flag beads and the precipitated proteins were detected with the indicated antibodies. (C) Colocalization of IFP35 and ORF6. HeLa cells were co‐transfected with IFP35‐Flag and GFP‐ORF6, and treated with BTZ for 8 h. Cells were then fixed, stained, and visualized by a confocal microscope (scale bar = 10 μm). A representative fluorescence intensity profile along the indicated line (white line) is shown. The fluorescency intensity and Pearson's correlation coefficients calculated from five randomly selected cells are shown on the right. (D) Schematic representations of IFP35 wild‐type and truncation mutants. FL, full length; L‐Zip, leucine zipper; NID, N‐myc‐interactor (NMI)/IFP35 domains. N, mutant containing the L‐Zip domain (1–80 aa); ΔC, C‐terminal deletion mutant lacking the NID2 domain (1–177 aa); M, mutant containing the NID1 domain (80–177 aa); ΔN, N‐terminal deletion mutant lacking the l‐Zip domain (80–288 aa); and C, mutant containing the NID2 domain (177–288 aa). (E) Requirement of both NID domains for the IFP35–ORF6 interaction. HEK293T cells were transfected with ORF6‐Flag and IFP35‐HA full‐length or the indicated truncation mutants for 24 h, followed by treatment with BTZ for 10 h. Whole‐cell lysates were immunoprecipitated with anti‐Flag beads and the precipitated proteins were detected with the indicated antibodies. (F) Critical role of the C‐terminal region of ORF6 in its interaction with IFP35. HEK293T cells were co‐transfected with IFP35‐Flag and ORF6‐HA or its mutants: M1 (49–52 aa substituted with alanines), M2 (53–55 aa substituted with alanines), and M3 (56–61 aa substituted with alanines) for 24 h, followed by treatment with BTZ for 10 h. Whole‐cell lysates were immunoprecipitated with anti‐HA beads and the precipitated proteins were detected with the indicated antibodies. The difference between the two groups was assessed with an unpaired, two‐tailed Student's *t* test. **p < 0.01.

### SARS‐CoV‐2 ORF6 inhibits IFP35‐induced GAS activation upon IFNγ treatment

Given the interaction between ORF6 and IFP35, we further investigated the inhibitory role of ORF6 on IFNγ signaling. We assessed how ORF6 affects IFP35 and found that, upon IFNγ stimulation, ORF6 overexpression reversed IFP35‐induced GAS activation and destabilized IFP35 at the protein level (Figure 4A). Consistently, the IFP35‐mediated upregulation of IFNγ‐stimulated genes, such as the genes *IRF1*, *CXCL9*, and *CXCL10*, could be abolished by ectopic expression of ORF6 (Figure 4B).

**Figure 4:**
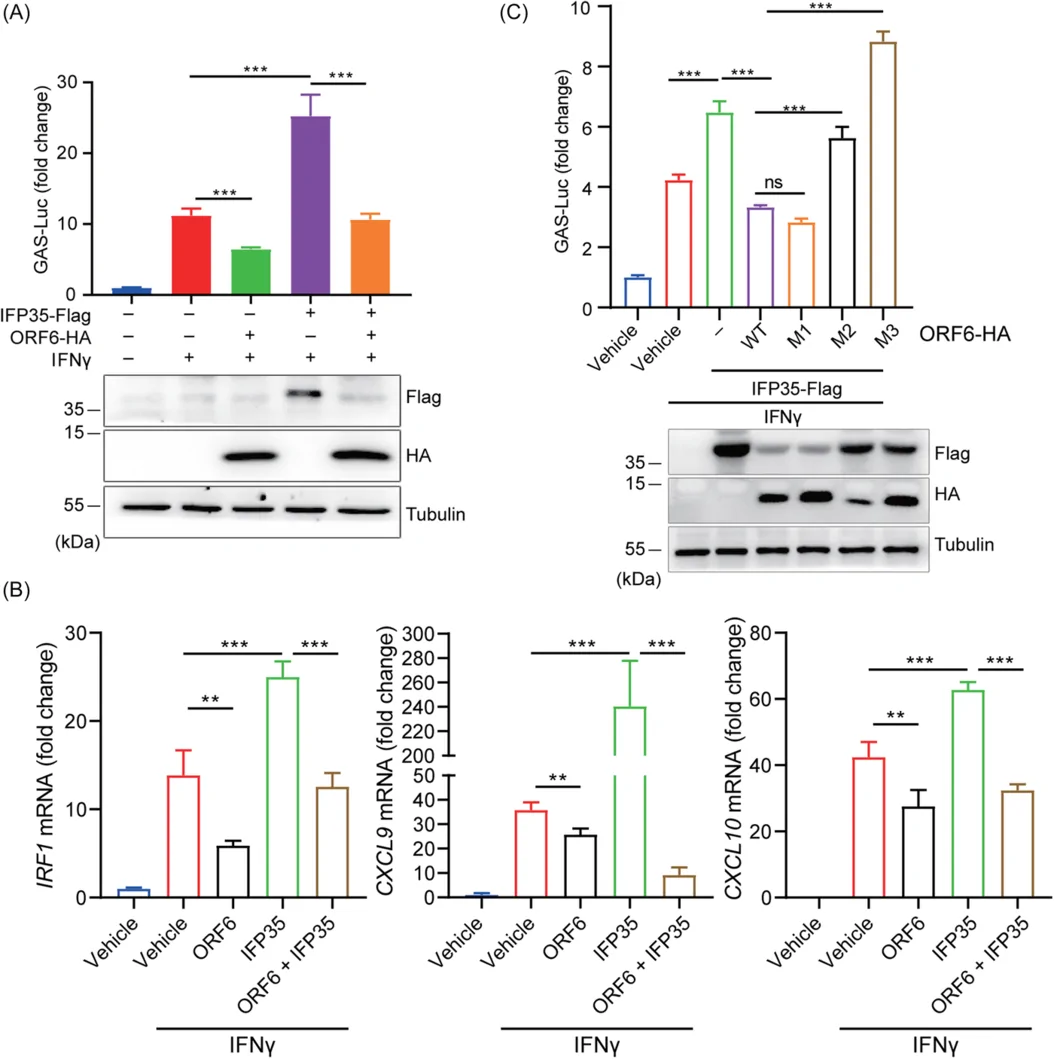
ORF6 inhibits IFP35‐induced GAS activation upon IFNγ treatment. (A) ORF6 destabilizes IFP35 and reverses IFP35‐induced GAS activation. HeLa cells were transfected with IFP35‐Flag and ORF6‐HA for 24 h, followed by treatment with IFNγ for 10 h. GAS‐Luc activity was then analyzed by the dual luciferase reporter assay, and protein expression levels were analyzed by immunoblotting. (B) ORF6 abolishes IFP35‐mediated upregulation of IFNγ‐stimulated genes. HeLa cells were transfected with the indicated plasmids and treated with IFNγ. The mRNA levels of the indicated genes were analyzed by RT‐qPCR. (C) The C‐terminal region of ORF6 is essential for IFP35 downregulation and GAS antagonism upon IFNγ stimulation. HeLa cells were transfected with IFP35‐Flag and ORF6 mutation plasmids: M1 (49–52 aa substituted with alanines), M2 (53–55 aa substituted with alanines), and M3 (56–61 aa substituted with alanines) for 24 h, followed by treatment with IFNγ for 10 h. GAS‐Luc activity was then analyzed by the dual luciferase reporter assay, and protein expression levels were analyzed by immunoblotting. Data represent mean ± SD of three independent experiments. All comparisons were made with one‐way ANOVA. **p < 0.01; ***p < 0.001; ns, not significant.

We further evaluated the influence of SARS‐CoV‐2 ORF6 C‐terminal mutants (Figure S2) on IFP35‐mediated GAS‐Luc activity, which is crucial for the antagonistic activity of ORF6 in host innate immune response^15^, ^16^. The results showed that both wild‐type ORF6 and ORF6 mutant M1 inhibited IFP35‐induced GAS promoter activation, whereas ORF6‐M2 showed a minimal inhibitory effect and ORF6‐M3 failed to exert inhibition (Figure 4C, top panel). Consistent with these findings, immunoblotting analysis revealed that ORF6‐M1 retained its ability to reduce the protein level of IFP35, while ORF6‐M2 and ORF6‐M3 showed a partial reduction (Figure 4C, bottom panel). Together, these findings demonstrate that SARS‐CoV‐2 ORF6 antagonizes IFP35‐induced GAS promoter activation upon IFNγ stimulation. The C‐terminal region spanning residues 53–61 is critical for both downregulating IFP35 protein levels and mediating its inhibitory effects.

### SARS‐CoV‐2 ORF6 promotes the proteasome degradation of IFP35

To investigate how ORF6 inhibits IFP35‐induced IFNγ activation, we assessed whether ORF6 affects the expression of IFP35. Compared to the empty‐vector control, the protein level of IFP35 was significantly decreased in cells overexpressing ORF6 (Figure 5A). Moreover, this reduction in the IFP35 level was dose‐dependent, suggesting that ORF6 decreases the protein level of IFP35 in cells. The observed decrease in the protein level could result from either downregulation of mRNA transcription or accelerated protein degradation. However, RT‐qPCR analysis showed that the mRNA abundance of IFP35 was not influenced by ORF6 overexpression compared with the vector control (Figure 5B). We next examined whether the ability of ORF6 to downregulate IFP35 depends on specific domains of IFP35. As shown in Figure 5C, co‐expression of ORF6 markedly reduced the protein levels of wild‐type IFP35, as well as various IFP35 deletion mutants (the ΔC, M, ΔN, and C mutants). In contrast, the IFP35 ΔNID1/NID2 mutant (the N mutant), which lacks both NID domains and fails to interact with ORF6 (Figure 3E), was resistant to ORF6‐mediated downregulation. These findings suggest that a physical interaction between ORF6 and the NID domains of IFP35 is required for ORF6‐induced degradation of IFP35. To determine the degradation pathway, we treated cells transfected with IFP35 and ORF6 with bortezomib (BTZ, a proteasome inhibitor) and bafilomycin A1 (BAF, an autophagy inhibitor). The results showed that only BTZ treatment significantly restored IFP35 levels (Figure 5D,E). Next, we measured the half‐life of IFP35 in cells with or without ORF6 expression using a cycloheximide (CHX) chase assay. In cells expressing ORF6, the half‐life of IFP35 was reduced to less than 12 h post‐CHX treatment, indicating that ORF6 enhances the degradation rate of IFP35 (Figure 5F). Additionally, ORF6 overexpression significantly increased the ubiquitination of IFP35 (Figure 5G). These findings suggest that ORF6 triggers the proteasome‐dependent degradation of IFP35 through ubiquitination.

**Figure 5:**
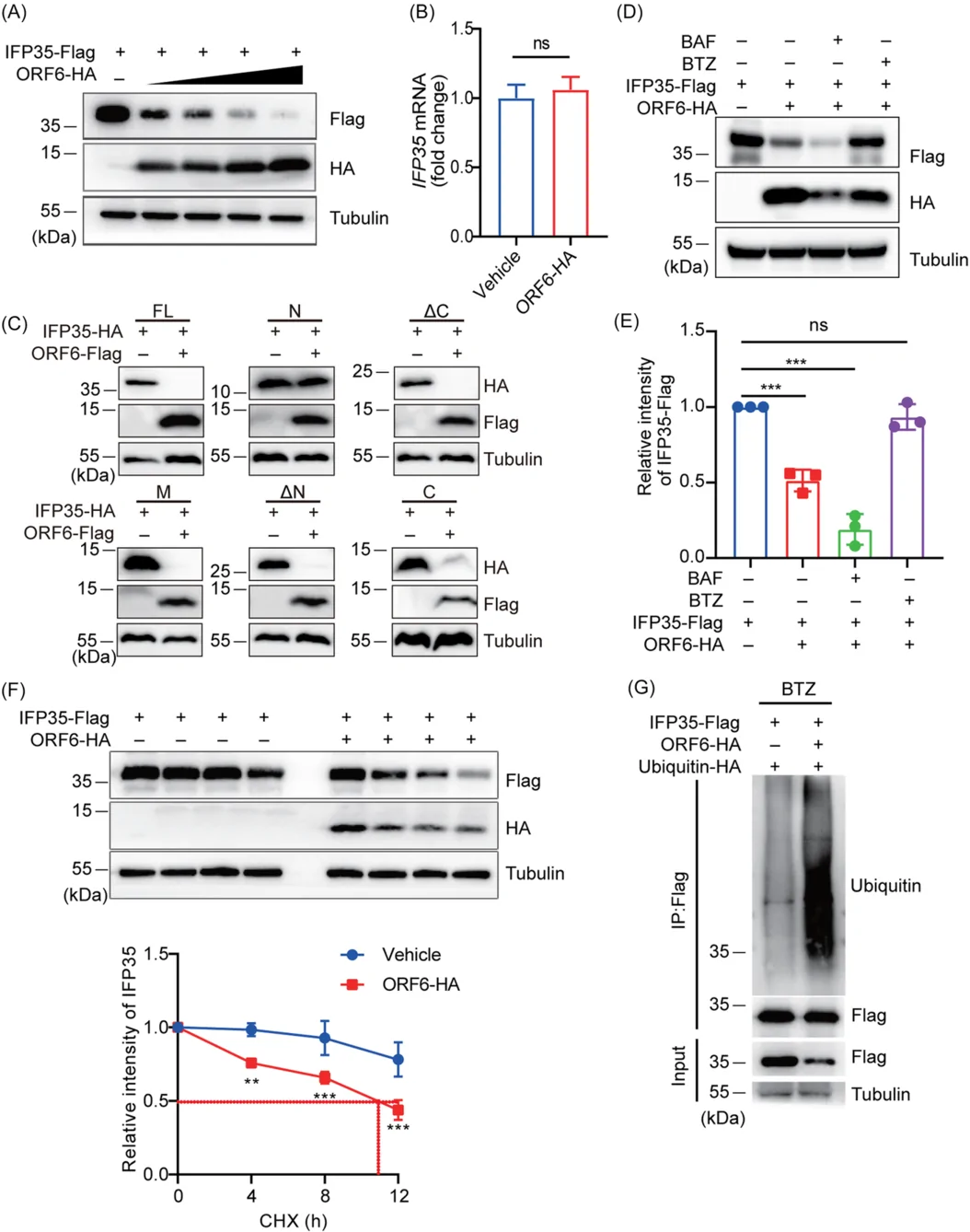
ORF6 promotes proteasome‐mediated degradation of IFP35. (A) ORF6 overexpression reduces IFP35 protein levels in a dose‐dependent manner. HEK293T cells were co‐transfected with 2 µg of the IFP35‐Flag plasmid and increasing doses of the ORF6‐HA plasmid (0, 0.5, 1, 1.5, and 2 µg) for 24 h. Protein expression was then analyzed by immunoblotting. (B) The mRNA level of *IFP35* is not influenced by ORF6 overexpression. HeLa cells were transfected with the ORF6‐HA plasmid or the corresponding empty vector and cultured for 24 h. Total RNA was then extracted and quantified by RT‐qPCR. *IFP35* mRNA levels were normalized to *GAPDH*. (C) The NID domains are required for ORF6‐mediated downregulation of IFP35. HEK293T cells were co‐transfected with IFP35‐HA (full‐length or the indicated truncation mutants) together with either the empty vector or ORF6‐Flag for 36 h. Cell lysates were then analyzed by immunoblotting using the indicated antibodies. FL, full length; N, mutant containing the L‐Zip domain (1–80 aa); ΔC, C‐terminal deletion mutant lacking the NID2 domain (1–177 aa); M, mutant containing the NID1 domain (80–177 aa); ΔN, N‐terminal deletion mutant lacking the L‐Zip domain (80–288 aa); and C, mutant containing the NID2 domain (177–288 aa). (D, E) Proteasome inhibitor BTZ restores IFP35 protein levels by immunoblot (D) and quantitative analyses (E). HEK293T cells were co‐transfected with 1 µg of IFP35‐Flag or with 0.5 µg of ORF6‐HA for 12 h, followed by treatment with BTZ or bafilomycin A1 (BAF) for 12 h. The protein levels were then analyzed by immunoblotting. Quantification of relative IFP35‐Flag protein levels from three independent experiments as shown in (D). (F) ORF6 mediates acceleration of IFP35 degradation. HEK293T cells were transfected with 2 µg of the IFP35‐Flag plasmid with or without 1 µg of ORF6‐HA for 24 h, followed by cycloheximide (CHX) treatment. Cells were harvested at the indicated time points (0, 4, 8, and 12 h post CHX treatment). Whole‐cell lysates were then analyzed by immunoblotting. (G) ORF6 overexpression increases the ubiquitylation of IFP35. HEK293T cells were co‐transfected with plasmids expressing IFP35‐Flag and Ubiquitin‐HA (Ub‐HA), together with either an empty vector or ORF6‐HA. At 24 h post transfection, cells were treated with BTZ for 8 h. Cells were then harvested, and whole‐cell lysates were subjected to immunoprecipitation using the anti‐FLAG® M2 Affinity gel. The immunoprecipitated proteins were analyzed by immunoblotting with the indicated antibodies. Ubiquitin levels were detected using an antiubiquitin antibody. Data represent mean ± SD of three independent experiments. Comparisons in (B) were made with an unpaired, two‐tailed Student's *t* test, those in (E) with one‐way ANOVA, and those in (F) with two‐way ANOVA. **p < 0.01; ***p < 0.001; ns, not significant.

## DISCUSSION

It is demonstrated in this work that IFNγ signaling is inhibited by the SARS‐CoV‐2 accessory protein ORF6 via downregulation of IFP35 (Figures 4 and 5). Our results showed that IFP35 activates the IFNγ‐mediated antiviral gene expression (Figure 1D, E), suggesting its role in enhancing IFNγ‐regulated antiviral immunity. SARS‐CoV‐2 ORF6 expression leads to IFP35 degradation and inhibits IFP35‐induced IFNγ activation, thereby helping the virus evade host immunity.

IFP35 is a cytosolic leucine zipper protein whose expression is induced by interferons and is highly conserved across species; however, its biological function remains incompletely characterized^26^. Previous studies have indicated that IFP35 functions in both intracellular transduction of IFN‐associated signaling cascades and extracellular secretion as a proinflammatory molecule capable of driving innate immune responses^27^, ^28^. Upon virus invasion, host cells rapidly activate their innate antiviral defense mechanisms. Several studies have highlighted that IFP35, as an ISG protein, plays a protective role against both viral and bacterial pathogens^29^, ^30^, ^31^. For instance, during chronic human hepatitis C virus (HCV) or human immunodeficiency virus (HIV) infection, IFP35 and other ISGs serve as IFN‐induced pro‐host factors that help inhibit viral replication^29^. In acute viral infections, the knockdown of *IFP35* expression leads to impaired type I interferon promoter activation and enhanced viral replication^30^. In this study, we found that SARS‐CoV‐2 infection upregulated *IFP35* in cultured cells and in mice. Furthermore, *IFP35* knockdown was found to markedly facilitate viral replication, indicating its antiviral role against SARS‐CoV‐2.

It has been further demonstrated that IFP35 enhances IFNγ‐mediated GAS reporter activity and downstream gene expression (Figure 1D, E) without affecting STAT1 phosphorylation or nuclear translocation (Figure S1E, F), suggesting its involvement in the downstream regulation of STAT1 signaling. It was reported that its related family member NMI, which shares structural homology with IFP35 (particularly in the NID domains), augments IFNγ‐dependent transcription by enhancing STAT1–CREB‐binding protein (CBP)/p300 complex formation^23^. It is therefore plausible that IFP35 similarly modulates the transcriptional activity of nuclear STAT1 complexes, potentially by recruiting or stabilizing cofactors at GAS‐containing promoters. Future mechanistic studies are needed to clarify how IFP35 augments IFNγ signaling.

SARS‐CoV‐2 has evolved diverse mechanisms to suppress host antiviral response, including those mediated by type I, II, and III IFNs, to successfully replicate in host cells^32^. While accessory proteins are dispensable for viral replication, they serve critical functions in modulating host immune antagonism and contributing to disease pathogenesis. ORF3a, ORF6, ORF8, and ORF9b have been documented to inhibit IFN production by disrupting various immune pathways^16^, ^17^, ^33^. Among these, ORF6 stands out as a potent IFN antagonist that impairs nuclear import of key transcription factors including STAT1 and IRF3. This impairment arises from its disruption of the karyopherin‐dependent nuclear import machinery^15^, ^16^. Additionally, ORF6 has been shown to suppress type II IFN‐mediated STAT1 signaling^20^. Our results are consistent with earlier studies implicating ORF6 in disrupting antiviral immune responses. However, we report a novel mechanism by which ORF6 antagonizes IFNγ‐mediated antiviral activity. Specifically, ORF6 interacts with IFP35 and promotes its proteasomal degradation via ubiquitination (Figures 3A‐F and 5G), thus inhibiting the IFNγ‐mediated signaling pathway (Figure 6). Interestingly, the C‐terminal of ORF6, which is crucial for blocking nuclear import, is also essential for ORF6‐mediated IFP35 degradation (Figures 3F and 4C), highlighting the importance of this region for the efficient anti‐IFN activity of ORF6.

**Figure 6:**
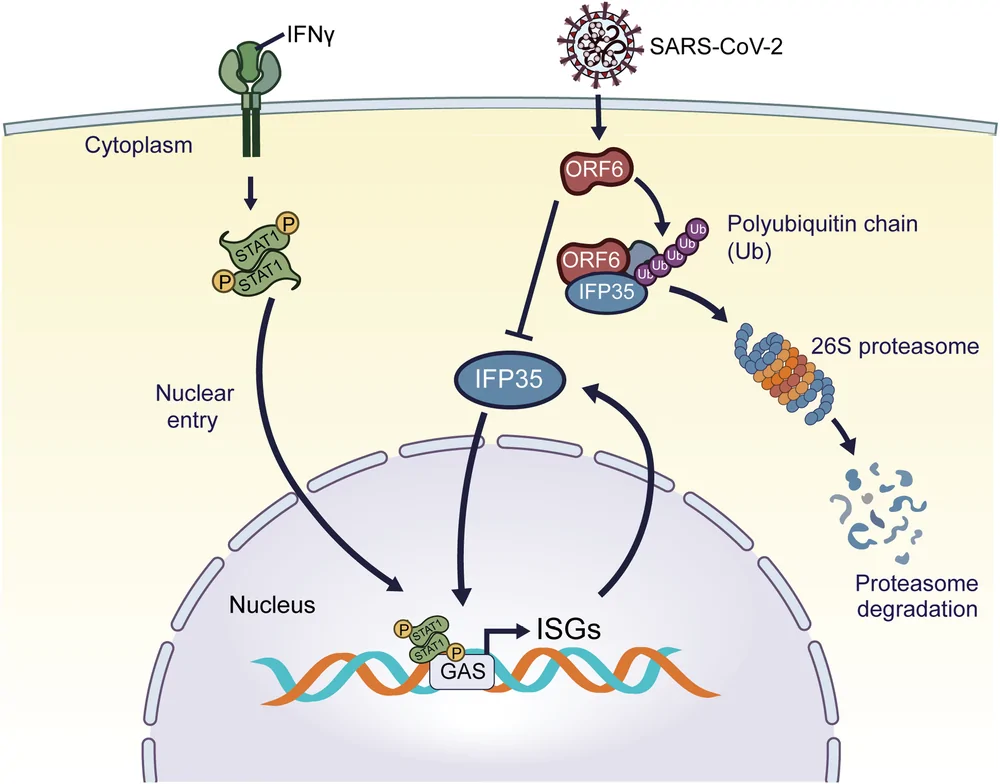
SARS‐CoV‐2 ORF6 suppresses IFNγ signaling by promoting ubiquitin‐proteasomal degradation of IFP35. Upon SARS‐CoV‐2 infection, the viral accessory protein ORF6 directly interacts with IFP35, targeting it for ubiquitin‐proteasomal degradation. This impairs IFP35's ability to activate IFNγ signaling, thereby attenuating the host antiviral response and facilitating viral replication. ISGs, interferon‐stimulated genes.

Our findings illustrate a dynamic host–virus conflict centered on IFP35. Upon SARS‐CoV‐2 infection, host antiviral defense is triggered, leading to the transcriptional upregulation of *IFP35* mRNA as part of the augmented IFNγ response. Concurrently, the virus deploys ORF6 to counteract this defense by binding IFP35 and promoting its ubiquitin‐proteasomal degradation, thereby blunting the downstream IFNγ signal. This interplay highlights a precise immune evasion strategy wherein the virus targets a host‐induced antiviral effector at the protein level to facilitate its replication.

Collectively, our data offer a fresh perspective on SARS‐CoV‐2 immune evasion, demonstrating that ORF6‐mediated dampening of IFP35 impairs IFNγ signaling. These findings deepen our understanding of how SARS‐CoV‐2 antagonizes host antiviral defenses and may facilitate the identification of potential therapeutic targets for COVID‐19.

## MATERIALS AND METHODS

### Re‐analysis of RNA‐seq data

Publicly available RNA sequencing data (GSE147507 and GSE150316) were downloaded from the NCBI Gene Expression Omnibus (GEO). After quality filtering to remove genes lacking detectable expression across all samples, raw counts were then normalized to counts per million (CPM) using edgeR's cpm() function in R, applying the formula CPM = (gene read count/total effective reads per sample) × 10^6^. To ensure data reliability, only genes expressed above CPM = 1 in ≥3 samples were retained for further processing.

To evaluate *IFP35* expression and the enrichment of relevant pathways in cells infected with SARS‐CoV‐2, we re‐analyzed RNA‐seq data from infected Calu‐3 and A549‐ACE2 cell lines (GSE147507)^8^. Normalized gene expression levels were expressed as CPM following trimmed mean of M‐values (TMM) normalization, using the edgeR's cpm() function. *IFP35* expression levels were then extracted for comparison across infection and mock conditions. Functional enrichment was assessed via Gene Set Enrichment Analysis (GSEA) executed through the clusterProfiler package (v4.14.6). Genes were ranked according to their log_2_ fold change (log_2_FC) values calculated from the differential expression analysis. Gene sets were extracted from the Molecular Signatures Database (MSigDB), via the msigdbr package, specifically focusing on the Hallmark (H) and Curated (C2) gene sets for *Homo sapiens*. Statistical significance was determined using 10,000 permutations, with results considered significant if *p* < 0.05. Visualization of the enrichment score (ES) profiles was generated using the enrichplot package.

To evaluate *IFP35* expression in lung tissues from SARS‐CoV‐2‐infected patients, the RNA‐seq dataset GSE150316, derived from autopsy lung specimens of patients who died from COVID‐19^22^, was re‐analyzed. Based on the quantitative RNA‐ISH assessment reported in the original study, patients with ≥2% virus‐infected lung area were classified as “Virus high” (11 cases) and those with <2% were classified as “Virus low” (12 cases). For the present analysis, we selected lung samples from 6 "Virus high" cases (*n* = 19 samples), 11 "Virus low" cases (*n* = 33 samples), and 5 non‐COVID‐19 negative controls, for which total RNA‐seq data were available. Raw sequencing data were processed and CPM calculation followed the same pipeline as that described above. *IFP35* expression levels were then extracted for comparison across groups.

### Cell culture and transfection

Cell lines including HEK293T, A549‐ACE2, HeLa, and Vero‐E6 were maintained in Dulbecco's modified Eagle's medium (DMEM) (11965118; Thermo Fisher) containing 10% FBS and 1% penicillin‐streptomycin. NCI‐H1299 cells were grown in RPMI 1640 (61870127; Thermo Fisher) with equivalent supplements. Cells were maintained at 37°C in 5% CO_2_. Transient transfection of HEK293T cells was performed using PEI (24765, Polysciences) at 2 µl PEI per 1 µg DNA. HeLa and NCI‐H1299 cells were transiently transfected via ExFect Transfection Reagent (T101‐01; Vazyme) using a pre‐optimized 1:2 (μg:μl) ratio.

### Antibodies and reagents

Antibodies utilized in this study included anti‐Flag (F1084; Sigma‐Aldrich), anti‐Beta‐Tubulin (100109‐MM05T; Sino Biological), anti‐HA (SAB1411738; Sigma‐Aldrich), anti‐Ubiquitin (10201‐2‐AP; Proteintech), anti‐IFP35 (83160‐4‐RR; Proteintech), anti‐STAT1 (14995T; CST), and anti‐pSTAT1(Tyr701, 9167T; CST). Recombinant human IFNγ (HY‐P7025A) was purchased from MCE. Recombinant human IFNβ (300‐02BC) was obtained from Thermo Fisher. The proteasome inhibitor bortezomib (BTZ, PS‐341, S1013) was purchased from Selleck. Bafilomycin A1 (BAF, 196000), an autophagy inhibitor, and cycloheximide (CHX, C0934), a protein synthesis inhibitor, were both sourced from Sigma‐Aldrich.

### Plasmids

The coding sequence for SARS‐CoV‐2 ORF6 was codon‐optimized and inserted into the pCAGGS expression vector, fused either with a C‐terminal Flag or an HA tag, or cloned into the pEGFP‐N1 backbone to generate the ORF6–GFP fusion construct. Site‐directed mutagenesis (Vazyme C214‐01) was utilized to generate three C‐terminal ORF6‐HA mutants (M1, M2, and M3), all of which were verified by Sanger sequencing. Human *IFP35* full‐length cDNA was subcloned into the pcDNA3.0 backbone to yield constructs with a C‐terminal Flag or an HA tag, and its truncation mutants with an HA tag were PCR‐amplified from the full‐length template. Three copies of the GAS were cloned into the pGL3.0‐basic vector to generate pGL3.0 GAS‐Luc. The ISRE‐Luc and pRL‐TK reporter plasmids were available in our laboratory. All plasmids were verified through sequencing.

### Virus and infection

The Delta variant of SARS‐CoV‐2 (B.1.617.2, GDPCC 2.00096) was sourced from the Guangdong Province Center for Disease Control and Prevention, having been isolated from clinical specimens collected from patients treated at Guangzhou Eighth People's Hospital. For cell infection, cultures were inoculated with the virus at a multiplicity of infection (MOI) of 0.05 in a serum‐free medium. The virus‐containing medium was removed after a 2‐h incubation and cells were washed before adding fresh DMEM supplemented with 2% FBS. Cultures were then maintained at 37°C under 5% CO_2_ for 48 h. All viral infection experiments were conducted within a biosafety level 3 (BSL‐3) facility at Guangzhou National Laboratory and Sun Yat‐sen University. Prior approval for all animal experiments was received from the Institutional Animal Welfare Committee, and all procedures were carried out in compliance with established ethical guidelines and institutional policies.

### Mice experiment

The SARS‐CoV‐2 infection studies in mice were conducted under animal biosafety level 3 (ABSL)‐3 conditions at Sun Yat‐sen University. K18‐hACE2 transgenic C57BL/6 mice (B6. Cg‐Tg (K18‐ACE2)2Prlmn/J) were assigned randomly to two groups (*n* = 3 each). Mice received either intranasal instillation of 500 plaque‐forming units (PFUs) of the SARS‐CoV‐2 Delta variant (lineage B.1.617.2) or an equivalent volume of PBS as a vehicle control. Three days after infection, animals were euthanized. Lung samples were homogenized in Dulbecco's phosphate‐buffered saline (DPBS), and the homogenates were clarified by centrifugation (3000 rpm, 10 min, 4°C). Clarified lysates were used for total RNA isolation and downstream RT‐qPCR quantification.

### RNA isolation and RT‐qPCR analysis

Total RNA was purified from samples utilizing TRIzol reagent (15596018; Invitrogen) in accordance with standard protocols. cDNA was synthesized from 1 µg of total RNA using the PrimeScript™ RT Master Mix (RR036A; TaKaRa). RT‐qPCR was subsequently carried out on a QuantStudio 5 Real‐Time PCR System (Applied Biosystems) with the PowerUp™ SYBR® Green Master Mix (A25742; Applied Biosystems). All assays were carried out in triplicate. The amplification program consisted of a 2‐min pre‐denaturation at 95°C, followed by 40 cycles of 95°C for 15 s and 60°C for 1 min. Gene expression values were normalized to *GAPDH* and quantified using the $2^{-∆∆C_{t}}$ method. The primer sequences are listed in Table S1.

### TCID_50_ assay

One day prior to infection, Vero‐E6 cells were plated into 96‐well plates. Supernatants harboring the virus were serially diluted 10‐fold in serum‐free DMEM. Aliquots of 100 μl from each dilution were applied to cell monolayers and incubated at 37°C for 1 h. After removing the inoculum, cells were washed once with DPBS. Subsequently, fresh prewarmed (37°C) DMEM containing 2% FBS (100 μl/well) was then added. After incubation for 48 h, the presence of the cytopathic effect (CPE) was assessed by microscopic examination. Viral titers were expressed as TCID_50_/ml determined using the Reed–Muench method.

### Dual‐luciferase reporter assay

The Dual‐Luciferase® Reporter (DLR®) assay system (E1910; Promega) was used to evaluate transcriptional activity. In brief, HeLa cells were seeded into 48‐well plates and transfected 24 h later with indicated plasmids, 50 ng of GAS‐Luc, and 10 ng of pRL‐TK plasmids per well. After 24 h of transfection, cultures were treated with 20 ng/ml IFNγ for 10 h. Cell lysates were then prepared and luciferase activities were measured using the Dual‐Glo® method as recommended by the manufacturer. Triplicate experiments were carried out for each condition. Results are expressed as the ratio of firefly luciferase activity to Renilla luciferase activity.

### Immunoprecipitation and immunoblotting

Transfected cells were harvested and lysed for 30 min on ice in IP buffer containing 50 mM Tris‐HCl (pH 7.4), 150 mM NaCl, 50 mM EDTA, 10 mM NaF, 10% glycerol, 1% Triton X‐100, and protease inhibitor cocktail (P8340, Sigma‐Aldrich). Centrifugation at 15,000*g* for 10 min at 4°C removed cell debris. The clarified lysates were then incubated with either anti‐FLAG® M2 Agarose Affinity Gel (A2220; Sigma‐Aldrich) or anti‐HA Magnetic agarose (SAE0197; Sigma‐Aldrich), and rotated at 4°C overnight. The affinity resins were washed thrice with IP buffer. Immunoprecipitates were released by boiling in 2× SDS‐PAGE loading buffer and resolved by SDS‐PAGE. Subsequently, proteins were transferred to PVDF membranes (ISEQ00010; Millipore) and blocked for 1 h at room temperature with 5% non‐fat milk in TBST. Then, the indicated primary antibodies were incubated overnight at 4°C. Following three washes with TBST, the appropriate HRP‐conjugated secondary antibodies were applied for 1 h at room temperature. After three washes, immunoreactive bands were detected using the Clarity™ Western ECL Substrate (#1705060; BioRad).

### Ubiquitination assay

The ubiquitination assay was conducted as follows: Plasmids encoding ubiquitin‐HA, IFP35‐Flag, and either ORF6‐HA or an empty vector were co‐transfected into HEK293T cells. After 24 h, cultures were treated with 1 μM BTZ for 8 h to inhibit proteasomal degradation and accumulate ubiquitinated proteins. Cells were then collected and lysed in IP buffer. Immunoprecipitation of IFP35‐Flag was carried out at 4°C overnight using the anti‐FLAG® M2 Agarose Gel (A2220; Sigma‐Aldrich). The immunoprecipitates were subsequently analyzed by immunoblotting: The anti‐Ubiquitin antibody detected ubiquitin conjugates, while the anti‐Flag antibody verified efficient IFP35 pulldown.

### Immunofluorescence staining

Cultured cells were seeded on glass coverslips in 12‐well plates and subjected to transfection the following day. After 24 h, cultures were exposed to 1 μM BTZ for 8 h. Fixation was achieved using 4% paraformaldehyde for 20 min at room temperature in the dark, then rinsed thrice with PBS, and permeabilized using 0.1% Triton X‐100. Blocking was conducted with 1% bovine serum albumin (BSA) for 60 min. The anti‐Flag antibody (1:500, F3165, Sigma‐Aldrich) was diluted in blocking buffer and incubated overnight at 4°C. Postwashing, the Alexa Fluor 555‐conjugated Donkey anti‐Mouse IgG secondary antibody (1:1000, A31570; Thermo Fisher) was applied for 1 h at room temperature in the absence of light. Nuclear staining was performed with DAPI (D1306; Invitrogen). Finally, slides were prepared by mounting the coverslips, and images were captured using a Carl Zeiss LSM880 confocal microscope with a 63× oil lens.

### RNA interference

siRNA‐mediated gene silencing was conducted using Lipofectamine RNAiMax Reagent (13778150; Thermo Fisher) following the instructions. Cells were plated in either 6‐ or 12‐well culture dishes and allowed to adhere overnight. When cells reached 50‐70% confluency, siRNAs targeting human IFP35 (siIFP35 #1: 5′‐GGAGCACACGAUCAACAUGTT‐3’; siIFP35 #2: 5′‐GCACACGAUCAACAUGGAGTT‐3’; siIFP35 #3: 5′‐ ACGUCCUGGAGAUCCACUUTT‐3′) or a nontargeting negative control were introduced. Specifically, RNAiMAX reagent and siRNA were diluted separately, followed by a 15‐min incubation to ensure complex formation. The complexes were subsequently added dropwise to the cells. At 48 or 72 h post transfection, knockdown efficiency was evaluated by RT‐qPCR.

### Statistical analysis

All statistical analyses were carried out using GraphPad Prism version 8.0 (GraphPad Software, Inc.). Results are summarized as mean ± standard deviation (SD), obtained from triplicate independent replicates. Pairwise comparisons were analyzed using an unpaired, two‐tailed Student's *t*‐test. For multi‐group comparisons, one‐way analysis of variance (ANOVA) was used. A *p*‐value cutoff of <0.05 was set, with significance marked as *p < 0.05, **p < 0.01, and ***p < 0.001.

## AUTHOR CONTRIBUTIONS

**Lihong Liu**: Methodology; project administration; validation; visualization; writing—original draft; writing—review and editing. **Jie Du**: Data curation; methodology; validation; writing—original draft. **Jiacheng Huang**: Methodology; validation. **Sidi Yang**: Methodology; writing—review and editing. **Qiaoqiao Xiao**: Resources. **Liu Cao**: Methodology. **Hong Peng**: Methodology. **Birong Zheng**: Methodology; resources. **Chunmei Li**: Writing—review and editing. **Deyin Guo**: Funding acquisition; project administration; supervision; writing—review and editing.

## ETHICS STATEMENT

Ethical approval for all animal studies was granted by the Sun Yat‐sen University's Institutional Animal Welfare Committee. All procedures were conducted in accordance with institutional animal care policies. Cell culture experiments involving live SARS‐CoV‐2 were conducted under BSL‐3 conditions, following approval from both the university's Institutional Biosafety Committee (IBC) and the Guangzhou National Laboratory. Sample inactivation was carried out in strict compliance with IBC‐validated standard operating procedures to ensure the safe transfer out of the ABSL‐3 facility.

## CONFLICT OF INTERESTS

The authors declare no conflict of interests.

## Supporting information

Supporting file 1.

## Data Availability

Supporting data of this study are accessible upon reasonable request from the corresponding author.

## References

[1] Chen Y , Liu Q , Guo D . Emerging coronaviruses: genome structure, replication, and pathogenesis. J Med Virol. 2020;92:2249. 10.1002/jmv.25681 PMC7167049 31967327

[2] Zhu N , Zhang D , Wang W , Li X , Yang B , Song J , et al. A novel coronavirus from patients with pneumonia in China, 2019. N Engl J Med. 2020;382:727–733. 10.1056/NEJMoa2001017 PMC7092803 31978945

[3] Wu F , Zhao S , Yu B , Chen YM , Wang W , Song ZG , et al. A new coronavirus associated with human respiratory disease in China. Nature. 2020;579:265–269. 10.1038/s41586-020-2008-3 PMC7094943 32015508

[4] WHO. WHO COVID‐19 dashboard. 2024. https://data.who.int/dashboards/covid19/cases?n=c

[5] Zhang X , Tan Y , Ling Y , Lu G , Liu F , Yi Z , et al. Viral and host factors related to the clinical outcome of COVID‐19. Nature. 2020;583:437–440. 10.1038/s41586-020-2355-0 32434211

[6] Hadjadj J , Yatim N , Barnabei L , Corneau A , Boussier J , Smith N , et al. Impaired type I interferon activity and inflammatory responses in severe COVID‐19 patients. Science. 2020;369:718–724. 10.1126/science.abc6027 PMC7402632 32661059

[7] Lokugamage KG , Hage A , de Vries M , Valero‐Jimenez AM , Schindewolf C , Dittmann M , et al. Type I interferon susceptibility distinguishes SARS‐CoV‐2 from SARS‐CoV. J Virol. 2020;94:e01410‐20. 10.1128/JVI.01410-20 PMC7654262 32938761

[8] Blanco‐Melo D , Nilsson‐Payant BE , Liu WC , Uhl S , Hoagland D , Møller R , et al. Imbalanced host response to SARS‐CoV‐2 drives development of COVID‐19. Cell. 2020;181:1036–1045.e9. 10.1016/j.cell.2020.04.026 PMC7227586 32416070

[9] Galani IE , Rovina N , Lampropoulou V , Triantafyllia V , Manioudaki M , Pavlos E , et al. Untuned antiviral immunity in COVID‐19 revealed by temporal type I/III interferon patterns and flu comparison. Nat Immunol. 2021;22:32–40. 10.1038/s41590-020-00840-x 33277638

[10] Ozgür A , Xiang Z , Radev DR , He Y . Literature‐based discovery of IFN‐gamma and vaccine‐mediated gene interaction networks. J Biomed Biotechnol. 2010;2010:426479. 10.1155/2010/426479 PMC2896678 20625487

[11] Boehm U , Klamp T , Groot M , Howard JC . Cellular responses to interferon‐γ. Annu Rev Immunol. 1997;15:749–795. 10.1146/annurev.immunol.15.1.749 9143706

[12] Busnadiego I , Fernbach S , Pohl MO , Karakus U , Huber M , Trkola A , et al. Antiviral activity of type I, II, and III interferons counterbalances ACE2 inducibility and restricts SARS‐CoV‐2. mBio. 2020;11:e01928‐20. 10.1128/mBio.01928-20 PMC7484541 32913009

[13] Sainz Jr. B , Mossel EC , Peters CJ , Garry RF . Interferon‐beta and interferon‐gamma synergistically inhibit the replication of severe acute respiratory syndrome‐associated coronavirus (SARS‐CoV). Virology. 2004;329:11–17. 10.1016/j.virol.2004.08.011 PMC7111895 15476870

[14] Rohaim MA , El Naggar RF , Clayton E , Munir M . Structural and functional insights into non‐structural proteins of coronaviruses. Microb Pathog. 2021;150:104641. 10.1016/j.micpath.2020.104641 PMC7682334 33242646

[15] Miorin L , Kehrer T , Sanchez‐Aparicio MT , Zhang K , Cohen P , Patel RS , et al. SARS‐CoV‐2 Orf6 hijacks Nup98 to block STAT nuclear import and antagonize interferon signaling. Proc Natl Acad Sci USA. 2020;117:28344–28354. 10.1073/pnas.2016650117 PMC7668094 33097660

[16] Lei X , Dong X , Ma R , Wang W , Xiao X , Tian Z , et al. Activation and evasion of type I interferon responses by SARS‐CoV‐2. Nat Commun. 2020;11:3810. 10.1038/s41467-020-17665-9 PMC7392898 32733001

[17] Xia H , Cao Z , Xie X , Zhang X , Chen JYC , Wang H , et al. Evasion of type I interferon by SARS‐CoV‐2. Cell Rep. 2020;33:108234. 10.1016/j.celrep.2020.108234 PMC7501843 32979938

[18] Krachmarova E , Petkov P , Lilkova E , Ilieva N , Rangelov M , Todorova N , et al. Insights into the SARS‐CoV‐2 ORF6 mechanism of action. Int J Mol Sci. 2023;24:11589. 10.3390/ijms241411589 PMC10380535 37511350

[19] Gioia U , Tavella S , Martínez‐Orellana P , Cicio G , Colliva A , Ceccon M , et al. SARS‐CoV‐2 infection induces DNA damage, through CHK1 degradation and impaired 53BP1 recruitment, and cellular senescence. Nat Cell Biol. 2023;25:550–564. 10.1038/s41556-023-01096-x PMC10104783 36894671

[20] Yoo JS , Sasaki M , Cho SX , Kasuga Y , Zhu B , Ouda R , et al. SARS‐CoV‐2 inhibits induction of the MHC class I pathway by targeting the STAT1‐IRF1‐NLRC5 axis. Nat Commun. 2021;12:6602. 10.1038/s41467-021-26910-8 PMC8594428 34782627

[21] De Masi R , Orlando S , Bagordo F , Grassi T . IFP35 is a relevant factor in innate immunity, multiple sclerosis, and other chronic inflammatory diseases: a review. Biology. 2021;10:1325. 10.3390/biology10121325 PMC8698480 34943240

[22] Desai N , Neyaz A , Szabolcs A , Shih AR , Chen JH , Thapar V , et al. Temporal and spatial heterogeneity of host response to SARS‐CoV‐2 pulmonary infection. Nat Commun. 2020;11:6319. 10.1038/s41467-020-20139-7 PMC7725958 33298930

[23] Zhu M , John S , Berg M , Leonard WJ . Functional association of Nmi with Stat5 and Stat1 in IL‐2‐ and IFNγ‐mediated signaling. Cell. 1999;96:121–130. 10.1016/s0092-8674(00)80965-4 9989503

[24] Cheng W , Chen S , Li R , Chen Y , Wang M , Guo D . Severe acute respiratory syndrome coronavirus protein 6 mediates ubiquitin‐dependent proteosomal degradation of N‐Myc (and STAT) interactor. Virol Sin. 2015;30:153–161. 10.1007/s12250-015-3581-8 PMC7091177 25907116

[25] Chen J , Shpall RL , Meyerdierks A , Hagemeier M , Böttger EC , Naumovski L . Interferon‐inducible Myc/STAT‐interacting protein Nmi associates with IFP35 into a high molecular mass complex and inhibits proteasome‐mediated degradation of IFP35. J Biol Chem. 2000;275:36278–36284. 10.1074/jbc.M006975200 10950963

[26] Bange FC , Vogel U , Flohr T , Kiekenbeck M , Denecke B , Böttger EC . IFP35 is an interferon‐induced leucine zipper protein that undergoes interferon‐regulated cellular redistribution. J Biol Chem. 1994;269:1091–1098. 8288566

[27] Yang W , Tan J , Liu R , Cui X , Ma Q , Geng Y , et al. Interferon‐γ upregulates expression of *IFP35* gene in HeLa cells via interferon regulatory factor‐1. PLoS One. 2012;7:e50932. 10.1371/journal.pone.0050932 PMC3514179 23226549

[28] Xiahou Z , Wang X , Shen J , Zhu X , Xu F , Hu R , et al. NMI and IFP35 serve as proinflammatory DAMPs during cellular infection and injury. Nat Commun. 2017;8:950. 10.1038/s41467-017-00930-9 PMC5643540 29038465

[29] Estrabaud E , Appourchaux K , Bièche I , Carrat F , Lapalus M , Lada O , et al. IFI35, mir‐99a and HCV genotype to predict sustained virological response to pegylated‐interferon plus ribavirin in chronic hepatitis C. PLoS One. 2015;10:e0121395. 10.1371/journal.pone.0121395 PMC4386819 25844942

[30] Zheng W , Li X , Wang J , Li X , Cao H , Wang Y , et al. A critical role of interferon‐induced protein IFP35 in the type I interferon response in cells induced by foot‐and‐mouth disease virus (FMDV) protein 2C. Arch Virol. 2014;159:2925–2935. 10.1007/s00705-014-2147-7 25085622

[31] Tan J , Qiao W , Wang J , Xu F , Li Y , Zhou J , et al. IFP35 is involved in the antiviral function of interferon by association with the viral tas transactivator of bovine foamy virus. J Virol. 2008;82:4275–4283. 10.1128/JVI.02249-07 PMC2293045 18305040

[32] Lowery SA , Sariol A , Perlman S . Innate immune and inflammatory responses to SARS‐CoV‐2: implications for COVID‐19. Cell Host Microbe. 2021;29:1052–1062. 10.1016/j.chom.2021.05.004 PMC8126603 34022154

[33] Wu J , Shi Y , Pan X , Wu S , Hou R , Zhang Y , et al. SARS‐CoV‐2 ORF9b inhibits RIG‐I‐MAVS antiviral signaling by interrupting K63‐linked ubiquitination of NEMO. Cell Rep. 2021;34:108761. 10.1016/j.celrep.2021.108761 PMC7857071 33567255

