# Supplementary material for: SARS‐CoV‐2 ORF6 protein targets IFP35 to inhibit the IFNγ signaling pathway
Source: mLife. 2026 Oct 26;5(5):597–610. doi: 10.1002/mlf2.70112 (PMC13710829; doi:10.1002/mlf2.70112)
Supplement: Supplementary file 1 [file MLF2-5-597-s001.docx]

**Supplementary Information**

**Supplementary Figures**

**Fig. S1** **T****he construction and verification of the pGL3.0-GAS-luc reporter, and the effect of IFP35 knockdown on STAT phosphorylation and nuclear translocation.** (A) Schematic representation of pGL3.0-GAS-luc reporter construct. (B) The reporter assay system was confirmed in HeLa cells treated with increasing doses of IFNγ. Luciferase activity was detected using the Dual Luciferase Reporter Assay. (C) HeLa cells were transfected with Flag-ORF6 and then treated with IFNγ. GAS-luc activity was detected using the Dual Luciferase Reporter Assay. (D) HeLa cells were transfected with increasing doses of IFP35-Flag for 24 h and then treated with IFNβ for 10 h. ISRE-luc activity was determined using the Dual Luciferase Reporter Assay. (E) HeLa cells were transfected with siIFP35 #1 and #3 for 48 h, followed by treatment with IFNγ for 1 h. Cell lysates were then analyzed by immunoblotting using the indicated antibodies. (F) HeLa cells were transfected with siIFP35 #1 and #3 for 48 h, followed by treatment with IFNγ for 1 h. Cell were then fixed, stained and visualized by confocal microscope (scale bar = 10 μm).

**Fig. S2 Schematic representation of SARS-CoV-2 ORF6 C-terminal variants. The expression of the mutant proteins was verified by immunoblotting.**

**Table S1. List of the primer sequences used for qRT- PCR.**

| **Gene** | **forward 5’-3’** | **reverse 5’-3’** |
| --- | --- | --- |
| *IFP35* | CACGATCAACATGGAGGAGTGC | GGCAGGAAATCCAGTGACCAAC |
| *IRF1* | GAGGAGGTGAAAGACCAGAGCA | TAGCATCTCGGCTGGACTTCGA |
| *CXCL9* | CCAACCAAGGGACTATCCACC | CCTTCACATCTGCTGAATCTGG |
| *CXCL10* | TGGCATTCAAGGAGTACCTC | TTGTAGCAATGATCTCAACACG |
| *GAPDH* | CGGAGTCAACGGATTTGGTC | GACAAGCTTCCCGTTCTCAG |
| SARS-CoV-2 *Sub-N* | CCAGGTAACAAACCAACCAA | TGAGTGAGAGCGGTGAACCAA |
